# Comparison of Gastric Emptying Time after the Ingestion of Whisky with Isocalorically Adjusted Glucose Solution

**DOI:** 10.1155/2022/6137230

**Published:** 2022-06-13

**Authors:** Tadashi Okabe, Hideo Terashima, Atsuhiro Sakamoto

**Affiliations:** ^1^Department of Anesthesiology, Hitachi Ltd., Hitachinaka General Hospital, 20-1 Ishikawa-cho Hitachinaka-shi, Ibaraki 312-0057, Japan; ^2^Department of Gastroenterological Surgery, Ibaraki Seinan Medical Center Hospital, 2190 Sakai-mati Sasima-gun, Ibaraki, Japan; ^3^Department of Anesthesiology, Nippon Medical School, Sendagi 1-1-5, Bunkyo-ku, Tokyo 113-8603, Japan

## Abstract

Previous studies have shown that the liquid gastric emptying mainly depended on energy content, regardless of compositional differences. But the gastric emptying of alcoholic beverages remains unclear. Therefore, we performed the present study to compare gastric emptying times between whisky mixed with water and glucose solution with uniform energy contents and volumes. As a crossover study, 10 healthy male volunteers ingested one of 3 test solutions with a uniform volume of 150 ml, i.e., whisky with water-containing whisky 30 ml (67 kcal), sugar water containing glucose 16.8 g (67 kcal), and water (0 kcal), and the gastric emptying time of each beverage was then assessed by ultrasound measurements of the gastric antral cross-sectional area. The gastric emptying pattern of whisky with water was faster than that of isocaloric sugar water, but slower than that of water. Each antral cross-sectional area 20, 30, and 40 min after the ingestion of sugar water was significantly larger than that of whisky with water. Antral cross-sectional areas 10 and 20 min after the ingestion of water were significantly smaller than those of whisky with water. In conclusion, the gastric emptying time of whisky would be faster than that of isocaloric glucose solution and slower than that of water. Unlike the other beverages, the gastric emptying time of alcohol drinks does not purely depend on the energy content because alcohol itself has no calorie before absorption. This study is registered with the University Hospital Medical Information Network (UMIN) Clinical Trials Registry (UMIN000034443).

## 1. Introduction

The current American Society of Anaesthesiologists (ASA) [1] and European Society of Anaesthesiology (ESA) [2] preoperative fasting guidelines recommend fasting from the intake of clear fluids for 2 hours or more before elective procedures requiring general anaesthesia. Although alcoholic beverages, such as whisky, beer, and wine, do not contain lipids, the ASA preoperative fasting guidelines state that “clear fluids” do not include alcohol; however, the underlying reasons are not stated [1]. The findings of studies on the effects of alcohol on gastric movement and/or emptying are not consistent [3–6], which has led to difficulties with clarifying the gastric emptying of alcoholic beverages on a theoretical basis. Previous studies indicated that the most significant factor influencing liquid gastric emptying was the calorie content of the fluid ingested [7–11]. We elucidate the underlying principle for liquid gastric emptying: gastric volume following the ingestion of liquids decreases exponentially over time [7, 8, 11]; under the condition that the initial volume of liquid ingested is fixed, the total amount of calories (calorie content) is a critical factor influencing liquid gastric emptying regardless of compositional differences, e.g., the ratio of fat to carbohydrates [7–11]; and the calorie content of the liquid ingested may be the primary factor influencing the gastric emptying rate rather than the volume [11]. When this underlying principle is applied to alcoholic beverages, i.e., whisky, the gastric emptying pattern of whisky with water is expected to be closer to that of water when their volumes are equal; whisky is noncaloric before being absorbed from the gastrointestinal tract because it only includes alcohol and none of the three major nutrients, namely, proteins, fats, and carbohydrates. The alcohol in whisky only acts as a source of energy equivalent to approximately 7 kcal/g when being metabolized in the liver [12]. Thus, the aim of the present study is to clarify the gastric emptying pattern of whisky with water by comparison to those of isocalorically adjusted sugar water or plain water only and thereby obtain a clearer understanding of the liquid gastric emptying of alcoholic beverages.

## 2. Materials and Methods

The present study was registered with the University Hospital Medical Information Network (UMIN) Clinical Trials Registry (ref. UMIN000034443), and ethical approval was received from the Hitachi Ltd., Hitachi General Hospital Research Ethics Committee (approval number 2018–63).

Ten healthy young male subjects participated in the present study as volunteers. Subjects were limited to males because a previous study showed that gastric emptying was affected by gender, the phase of the menstrual cycle, and the menopausal status [13]. After obtaining written informed consent on the prescribed form, all 10 subjects were found to be free of medical conditions associated with delayed gastric emptying (e.g., diabetes, severe obesity, and gastric diseases) and alcohol intolerance through a questionnaire that included health history and physical activity. The present study was designed as a crossover study, but was not blinded for both the subjects and the evaluator who performed all ultrasound assessments because while the test solution was certainly discriminated by taste, some subjects showed characteristic reactions such as a drunk person after ingesting whisky with water.

The study protocol was designed based on the method described in our previous study [7]. According to the recommendations for preoperative fasting before general anaesthesia [1, 2], the ingestion of solids was stopped 6 hours before and clear fluids 2 hours before the examination. A subject ingested one of the following 3 types of test solutions stored at room temperature, over the course of approximately 3 min. After ingesting any one of the 3 types, subjects maintained a seated position at a 45° angle and underwent an ultrasound examination every 10 min up to 120 min after the beverage had been ingested. An ultrasound assessment to calculate the cross-sectional area of the gastric antrum was performed in the right lateral position. In order to ensure technical uniformity, all measurements were performed by only one evaluator (the lead author of this study). The same subject consumed another one of the 3 test solutions on a different day (there was no clearly defined test interval) and underwent the same ultrasound examination. This pattern was repeated until the 3 different test solutions had been ingested in subsequent examinations. The following 3 types of test solutions with a uniform volume were prepared:

1. 150 ml of whisky with water (30 ml of whisky-containing 40% alcohol + 120 ml of water): whisky group
2. 150 ml of sugar water (11.2% glucose solution): sugar group
3. 150 ml of water alone: water group

Whisky with water and that with sugar water were both isocalorically adjusted to 67 kcal. In the whisky group, alcohol intake was equal to that of a so-called “shot of whisky.”

In ultrasound imaging, the gastric antrum was identified in the sagittal to right parasagittal plane using the left lobe of the liver, the pancreas, abdominal aorta, or inferior vena cava as anatomical landmarks, as described previously [14–17]. When the minimal antral cross-sectional area of the gastric antrum was imaged using ultrasonography (SonoSite S II, FUJIFILM SonoSite, Inc., Bothell, WA, USA), the anteroposterior (AP) and craniocaudal (CC) diameters were both measured and recorded in the database, as described previously [14–17].

The following formula was used to calculate the antral cross-sectional area (CSA).

(1) $$\begin{array}{c} \text{Antral CSA}=\frac{\pi \times \text{AP}\left(cm\right)\times \text{CC}\left(cm\right)}{4}. \end{array}$$

Gastric volume was calculated from the gastric antral cross-sectional area, as described previously [14].

(2) $$\begin{array}{c} \text{Gastric volume}=27+14.6\times \text{CSA}\left({\text{cm}}^{2}\right)-1.28\times \text{age}\left(\text{year}\right). \end{array}$$

A graph of the antral cross-sectional area was made for each group via box-and-whisker plots. A gastric emptying curve was made by plotting the median values of gastric volumes at each measurement point. Nonparametric methods were used since the data did not show a normal distribution. A statistical comparison of three groups was performed with the Friedman test. When significant differences were noted, multiple comparisons between the whisky group and sugar or water group were made using the Wilcoxon signed-rank sum test with the Bonferroni correction. A *P* value of <0.05 was considered to be significant. All statistical analyses were conducted using Excel statistical program file Ystat 2013 (developed by Yamazaki S, Igakutosyo Syuppan Co., Ltd., Tokyo, Japan).

## 3. Results

**Figure 1:**
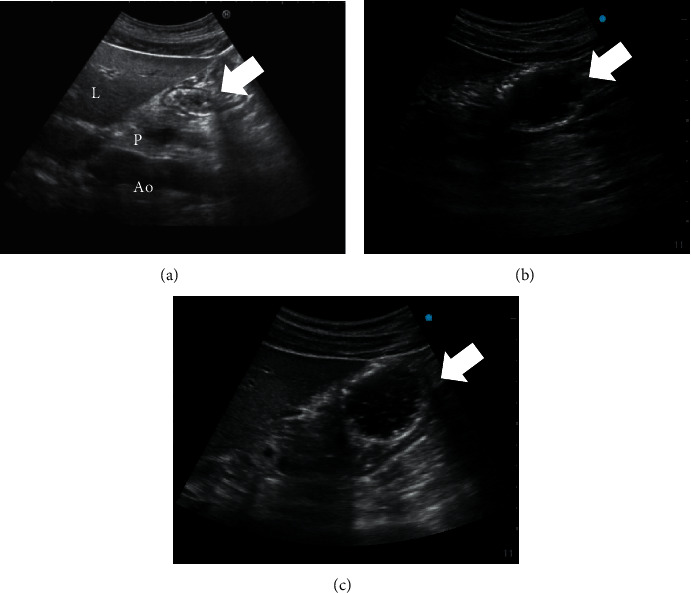
(a) Sagittal sonogram of the empty gastric antrum. The antrum is located between the left lobe of the liver and the pancreas at the level of the aorta or inferior vena cava. (b) Sagittal sonogram of the gastric antrum 10 minutes after the ingestion of 150 ml of diluted whisky (30 ml of whisky + 120 ml of water). The antrum appeared distended with a hypoechoic fluid content. (c) Sagittal sonogram of the gastric antrum 10 minutes after the ingestion of 150 ml of 12.5% glucose solution. The antrum appeared distended with hypoechoic fluid. Arrow, antrum; L, liver; P, pancreas; Ao, aorta.

Ten healthy male participants consented to, enrolled in, and completed the present study. The following data on their physical characteristics are shown as means and standard deviations (SD); age 32.4 (5.9) years, height 171.2 (5.2) cm, weight 68.4 (8.8) kg, and body mass index 23.0 (2.4) kg·m^2^.  Figure 1 shows the typical ultrasound images obtained during this study.

**Figure 2:**
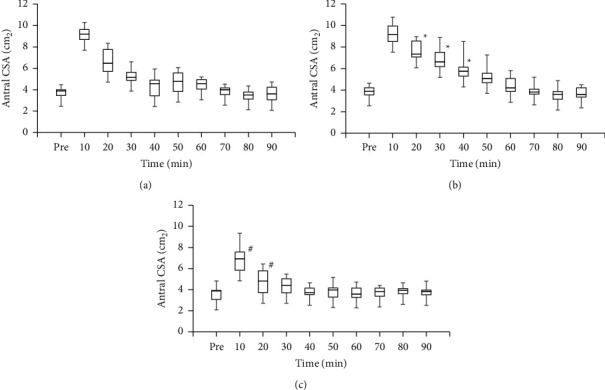
(a) Changes in gastric antral cross-sectional areas (CSA) over time in the whisky group. (b) Changes in gastric antral CSA over time in the sugar group. (c) Changes in gastric antral CSA over time in the water group. Each data point is presented as a box-and-whisker plot. The box denotes the first and third quartiles, with the whiskers representing the range. The median is indicated by a line. Whisky group, 150 ml of diluted whisky (30 ml of whisky + 120 ml of water, 67 kcal); sugar group, 150 ml of 12.5% glucose solution (67 kcal); water group, 150 ml of water (0 kcal). ^*∗*^*P* < 0.05, significant difference between the whisky group and sugar group by the Wilcoxon signed-rank sum test with Bonferroni corrections; ^#^*P* < 0.05, significant difference between the whisky group and water group by the Wilcoxon signed-rank sum test with Bonferroni corrections.

**Figure 3:**
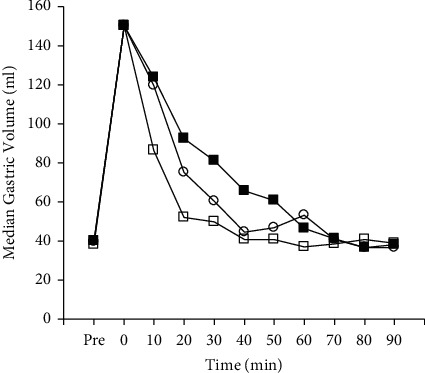
Changes in medians of calculated gastric volumes over time after the ingestion of one of 3 different beverages. Whisky group (◯), 150 ml of diluted whisky (30 ml of whisky + 120 ml of water, 67 kcal); sugar group (■), 150 ml of 12.5% glucose solution (67 kcal); water group (□), 150 ml of water (0 kcal).

Using a plot box, Figure 2 shows changes in the gastric antral cross-sectional area over time in each group, i.e., the whisky group, sugar group, and water group. In the Friedman test, significant differences were observed among the groups (10, 20, 30, and 40 min after ingestion; *P* < 0.01). Antral CSA 20, 30, and 40 min after ingestion were significantly larger in the sugar group than in the whisky group (*P* < 0.05; the Wilcoxon signed-rank sum test with Bonferroni correction; *n* = 10; Figure 2(b)). Antral CSA 10 and 20 min after ingestion were significantly smaller in the water group than in the whisky group (*P* < 0.05; the Wilcoxon signed-rank sum test with Bonferroni correction; *n* = 10; Figure 2(c).  Figure 3 shows the medians of the calculated gastric volumes in each group were simply plotted over time for the purpose of showing an overview suggesting that while each gastric emptying pattern exhibited an exponential manner, the gastric volume in the whisky group decreased faster than in the sugar group but slower than in the water group.

## 4. Discussion

In the present study, the gastric emptying time of whisky with water was faster than that of the same volume of the isocaloric glucose solution, as anticipated, but was slower than that of the same volume of water, indicating that some factors present in whisky delayed emptying.

Whisky includes only alcohol (ethanol) and none of the three major nutrients, namely, proteins, fats, and carbohydrates. Therefore, whisky may be nutritionally deemed a calorie-free beverage before being absorbed from the gastrointestinal tract. Alcohol acts as a source of energy equivalent to approximately 7 kcal/g only when it is metabolized in the liver. In the liver, alcohol is metabolized in the order of acetaldehyde, acetate, and the end products of water and carbon dioxide. Since acetate derived from this metabolic process is quickly utilized in the tricarboxylic acid cycle, no metabolic products are stored as an energy source [12]. In contrast, brewed liquors, such as beer and wine, include nutrients that act as an energy source per se, in contrast to alcohol, and thus, each gastric emptying time is expected to depend on the calorie content. A previous study reported that the gastric emptying time of beer and red wine was significantly longer than that of ethanol under the condition that volumes and alcoholic contents were both uniform, while that of beer, which has a lower calorie content, was shorter than that of wine [6].

Calorie intake is a regulator of the gastric emptying time. Previous studies demonstrated that liquid gastric emptying chiefly depended on the total amount of calories regardless of compositional differences [7–11]. The small intestine chiefly performs digestion and the absorption of water and nutrients, whereas the stomach breaks, stirs, and preserves ingested materials to promote smooth digestion and absorption in the small intestine. If large amounts of ingested nutrients are flowing into the small intestine at once, metabolic abnormalities, including hyperglycaemia, will occur. Dumping syndrome after gastrectomy is one of these pathological conditions. When nutrients are absorbed in the proximal and/or distal small intestine, various gastrointestinal hormones, such as secretin, gastric inhibitory peptide, cholecystokinin, glucagon-like peptides, and peptide YY, are secreted as a feedback mechanism, thereby allowing for the appropriate digestion and absorption of nutrients [18–21], which suppresses the motility of the stomach and upper jejunum. One of those feedback systems is called the “ileal brake” [21].

Based on these two standpoints, the gastric emptying pattern of whisky with water is expected be closer to that of water when adjusted to the same volume. In the present study, the gastric emptying time of whisky with water was significantly slower than that of water alone; therefore, the effects of alcohol need to be considered. Although abnormal motility, mucosal damage, and increased permeability occur in the digestive tract following the consumption of alcohol [3], gastric motility exhibits two very different patterns depending on the alcoholic content. Bujanda and co-workers showed that gastric motility was inhibited by the intake of alcoholic beverages with a density of more than 15%, but was promoted at lower densities [4]. Franke and co-workers reported that the gastric emptying times of ethanol adjusted to low alcoholic contents of 4 and 10% were longer than that of water, and no significant differences were observed in gastric emptying times in the range of 4–10%; however, the underlying mechanisms remain unclear [6]. In the present study, we used whisky with water corresponding to a low alcoholic content of 8%. Although gastric motility is expected to be facilitated by a low alcoholic content, the gastric emptying of whisky with water was significantly longer than that of water in the present study. The following two major factors may be contributing to this phenomenon. The stomach is one of the principal absorption sites of ingested alcohol and has the ability to metabolize ethanol by alcohol dehydrogenase (ADH), such as ADH1C, ADH3, and ADH4, despite a low metabolic capacity, suggesting that absorption and metabolic processes in the stomach induced delayed emptying against enhanced gastric motility [22, 23]. This insight may be important for the liquid gastric emptying of alcoholic beverages. Furthermore, the osmotic pressure of liquids is a factor that inhibits gastric emptying [24–26]. Previous studies have shown that differences in gastric emptying times due to osmotic pressure were markedly smaller than those due to the calorie content [25, 26]; however, a large difference in osmotic pressure significantly affects gastric emptying rates [24]. Since the osmotic pressure of ethanol is 165 mosm/L per 1% [6], the osmotic pressure of whisky with alcohol used in the present study was calculated to be at least 1320 mosm/L, which is more than 132-fold that of water (10 mosm/L). This large difference results in the slower emptying of whisky with water. Further studies that investigate other factors are required.

There were some limitations in the present study. The number of subjects (*n* = 10) examined was small, and all were physically and mentally healthy. Therefore, the results of the present study may not be applied to individuals with alcohol allergies or intolerances or patients including those receiving anaesthesia and undergoing surgery. Furthermore, since the number of subjects (*n* = 10) examined was small, the present study is a type of an exploratory study.

## 5. Conclusions

Under the condition of a uniform volume, the gastric emptying time of whisky with water was faster than that of an isocaloric glucose solution, but slower than that of water. The gastric emptying of whisky did not obey the underlying principle that the calorie content of an ingested liquid is the primary factor influencing the gastric emptying rate, which may be associated with the unique properties of whisky that differ from those of nonalcoholic beverages, i.e., the gastric absorption and metabolism of ethanol and high osmolality.

## Data Availability

The data used to support this study are available from the corresponding author upon request.
